# Double stigma: a cross-sectional study of Lassa patients with hearing loss in North Central Nigeria

**DOI:** 10.3389/fpubh.2024.1395939

**Published:** 2024-07-15

**Authors:** Kachollom C. Best, Emmanuel Ameh, Caroline Weldon, David Shwe, Ibrahim Mahmood Maigari, Ishaku Turaki, Nuhu D. Ma’an, Christopher Sabo Yilgwan, Tomoko Makishima, Scott Weaver, Slobodan Paessler, Nathan Y. Shehu

**Affiliations:** ^1^Department of Sociology, Faculty of Social Sciences, University of Jos, Jos, Nigeria; ^2^West African Center for Emerging Infectious Diseases, Jos University Teaching Hospital, Jos, Nigeria; ^3^Department of Microbiology, University of Texas Medical Branch, Galveston, TX, United States; ^4^Department of Paediatrics, Jos University Teaching Hospital, Jos, Nigeria; ^5^Department of Internal Medicine, Abubakar Tafawa Balewa University Teaching Hospital, Bauchi, Nigeria; ^6^Department of Otorhinolaryngology, Jos University Teaching Hospital, Jos, Nigeria; ^7^Department of Ear, Nose and Throat, University of Texas Medical Branch, Galveston, TX, United States; ^8^Department of Pathology, University of Texas Medical Branch, Galveston, TX, United States; ^9^Department of Internal Medicine, Jos University Teaching Hospital, Jos, Nigeria

**Keywords:** Lassa fever, hearing loss, stereotyping, stigma, apathy, survivors

## Abstract

**Introduction:**

Lassa fever is a zoonotic infectious disease endemic in West Africa with a high case-fatality rate and reported stigmatization of surviving patients. This study examines discrimination among survivors of Lassa fever (LF) complicated by hearing loss (HL).

**Methods:**

This cross-sectional qualitative study used an in-depth interview guide to collect information from patients with HL about their experience of stigma. Interviews were conducted by a trained team of interviewers at the Jos University Teaching Hospital between January and April 2022 in Hausa language after informed consent was obtained. Recordings of the interviews were transcribed and translated from Hausa to English. Data analysis was conducted using *NVivo* software using a thematic framework approach.

**Results:**

Most (73%) respondents were male (*n* = 11); 27% were female (*n* = 4). The median age was 35 years (interquartile range, 16.5). Some Lassa fever patients experienced stigma and discrimination (53%) including isolation and withdrawal of family and community support during and after illness. HL increased stigma, as some patients were labeled “deaf” by other community members, increasing perceived stigma and devaluation. HL affected the socio-economic wellbeing of some who could not communicate well with their families and customers and constrained social interactions, evoking pain and apathy. Some survivors of LF and victims of its sequelae of HL experienced double stigmatization. While they were ill with LF, a third of respondents reported avoidance and isolation by family and community members who withdrew care and support both to them and their close family members. These forms of stigmatization strained their relationships.

**Conclusion:**

There is a need to address stigma in LF survivors who develop HL through concerted community-owned awareness to improve their quality of life along with a robust social support system to aid prevention.

## 1 Introduction

Lassa fever (LF) is a zoonotic infectious disease transmitted to humans from multimammate rats (*Mastomys natalensis*). The first human case of LF was detected in Borno State, Nigeria, and LF continues to be a major public health concern with a 3–42% case-fatality rate in Nigeria (1). Outbreaks of LF occur most commonly in West and Central African countries (2–4). In addition to direct spill over infections from rats, nosocomial transmission occurs through contact with the bodily fluids of infected persons.

All social groups irrespective of age or sex are vulnerable to infection, and the case-fatality rate of LF in Africa among confirmed cases ranges from 15 to 20% (2, 4, 5); there is no uniform surveillance system for LF in the continent. The incidence of LF is reported to have increased in the last decade (6), and Nigeria has witnessed an increase in the rates of confirmed Lassa fever virus (LASV) infections during outbreaks from December 2016 to September 2020. In 2017, there were 298 confirmed cases, more than doubling to 528 in 2018, 796 in 2019, and 1,165 cases confirmed in 2020. The number of states in Nigeria with confirmed LF outbreaks surged from 19 in 2017 to 32 out of 36 states of the Federation and the Federal Capital Territory (FCT) in 2020 (4).

Although an association between LF and sudden onset sensorineural hearing loss has been confirmed clinically, the prevalence and mechanism of LF-induced hearing loss is still poorly understood. Approximately one-third of LF survivors develop sudden onset sensorineural hearing loss, and this constitutes a neglected public health and social burden (7). Lassa virus-induced immunological injury to the structures of the inner ear has been suggested as the initiating cause of hearing loss in LF (8). An animal study (in a murine model) has shown that the pathology in the inner ear consists of damage to the cochlear hair cells as well as degeneration of the spiral ganglion cells of the auditory nerve (9).

Symptomatic LF patients not only experience the consequences of ill health and economic costs of infection but also the psychosocial impacts. Stigma, as conceptualized by Goffman, is difference in the physical, mental, moral, or group identity of people who are judged differently and, on that basis, treated unfairly by those who share a common identity (10). Stigma follows certain health conditions, either infectious or non-infectious diseases, when it is understood that healthy people are at risk of contracting the same disease, or when the disease has high mortality rates, leading to fear, which may be driven by ignorance and misconception (11). People suffering from leprosy, tuberculosis, HIV infection/AIDS, and LF throughout the world encounter stigma and discrimination (12). Lassa fever, like most infectious diseases, evokes fear that mostly results in patients experiencing stigma from both the public and health professionals due to its high infectivity, and it can only be diagnosed after diagnostic tests are conducted, increasing the vulnerability of those that had contact with the patients before test results are available (13). A study in communities affected by LF in Ebonyi State, Nigeria found that approximately 32.2% of respondents expressed stigmatizing attitudes that they were unlikely to accept survivors who had been successfully treated (2). Even children whose parents were infected with LASV suffered stigma and discrimination as community members, including relatives, no longer welcomed them into their homes (14).

Stigma has been reported among those with hearing loss (15), despite being a non-infectious health problem. A third of LF survivors develop sequelae including hearing loss, as they experience a sudden onset of hearing loss (HL) which is either temporary or permanent (7). They have been found to suffer social exclusion and labelling in family and community life (14). Thus, a large portion of those infected with LASV may experience stigma like other patients with infectious diseases (16) and may also suffer double stigma from hearing loss. However, this has not been fully explored in the literature.

We therefore conducted a qualitative study to explore the nature and sources of double stigma both from LASV infection and its hearing loss sequelae and the impact of illness on those who experienced LASV infection complicated with HL. We also investigated the attitude and perceptions of participants about LF.

## 2 Methods

### 2.1 Background of the study area

Bauchi State has the second highest prevalence of LF in Northern Nigeria, accounting for 4.81% (*n* = 134) of the national total from December 2016 to September 2020 (4). Our study was conducted among LF survivors with hearing loss attending the Ear, Nose, and Throat (ENT) department of the Jos University Teaching Hospital (JUTH) from rural communities of Toro LGA (Local Government Area), as well as Toro (the Local Government headquarters), a peri-urban area in Bauchi State, Northern Nigeria. The preference of LF survivors for the study to be conducted at JUTH rather than their respective communities was indicative of the experiences of stigma motivating the study.

Thus, JUTH was used as the study center, based on patients’ preference, to interview them on their clinical visit days. The hospital serves as a referral center for hospitals within Plateau State as well as hospitals in neighboring states of Taraba, Bauchi, Gombe, Benue, Nasarawa, Kogi, and parts of Kaduna and the Federal Capital Territory (FCT), Abuja.

### 2.2 Study design

This cross-sectional study was conducted to gather qualitative data using an in-depth interview (IDI) guide from survivors of LF with complications of HL from Bauchi State at the ENT Unit. Interviews were lead and recorded by a JUTH research team in Hausa language, which were later translated into English. The study was conducted between January 4, 2022 and April 30, 2022.

### 2.3 Population and sample

The study population was survivors of LF with complications of HL. Participants were purposively selected from LF survivor patients attending follow up appointments at the Jos University Teaching Hospital ENT Clinic. The inclusion criteria were sensorineural hearing loss defined by hearing loss of pure tone average of at least 30 dB, abnormal function of the cochlea, auditory nerve, or higher aspects of central auditory perception or processing assessed with Auditory Brainstem Responses (ABR) and Distortion Product Otoacoustic Emissions (DPOAE) (17) and confirmation of Lassa fever by RT-PCR.

The exclusion criterion was the presence of hearing loss prior to Lassa fever infection. Participants were recruited and interviewed until data saturation was achieved when new information no longer brought fresh insights to the research questions. Fifteen IDIs were conducted with the patients or caregivers of children during the study period. The participants had no relationships with the interviewers before the study.

### 2.4 Data collection and data analysis

After written informed consent was obtained, an IDI guide was used to collect qualitative data from participants by face-to-face interviews until saturation was achieved. The interview guide was reviewed and adopted by reviewers. Interviews lasted between 30 and 45 min. No relationship was established between the interviewers and the participants before the study. The interviewers included a lecturer (Kachollom Best) with a PhD in sociology, a sociologist with an MSc in sociology, and a sociologist with a BSc in sociology. All interviewers were female and have years of research in the social sciences. Most of the interviews were conducted in the Hausa language and a few were performed in English and Hausa language. The interviews were transcribed to English by language transcription experts proficient in both Hausa and English language. Using a thematic framework approach, the transcripts were analyzed using *NVivo (Version 12; QSR International, Melbourne, Australia)* qualitative analysis software (18). A thematic coding framework was developed, arising from themes emerging from the data, original aims and objectives, and the topic guide, to identify emerging themes from the data related to health status at baseline, knowledge and perception of LF, stigma due to LF infection, stigma due to hearing loss, and prevention of LASV infections. Sections of text were extracted from the original transcripts, indexed and summarized based on their relevance to the identified themes.

### 2.5 Ethics statement

Ethical approval was obtained from the Jos University Teaching Hospital Research Ethics Committee (Approval number: JUTH/DCS/ADM127/XXVI/669 & NHREC/JUTH/05/1022). All procedures in this study were in conformity with the principles of the Helsinki Declaration. Written informed consent was obtained from all study participants.

## 3 Results

### 3.1 Demographic characteristics

IDIs were conducted for 15 patients, 11 of whom were males. The majority of participants (*n* = 11; 73.3%) were married, one was widowed due to LF, and three were single. The average age of the participants was 35 years (IQR, 16.5), very few (*n* = 3) had tertiary education and most had secondary level of education or less. Most participants (*n* = 8; 62%) were employed in the non-formal sector as petty traders or subsistence farmers, which is generally characterized by low income and low farm yields. Approximately two-thirds (*n* = 7; 63.64%) lived in large households of 8–15 people. Our participants did not have health insurance cover; therefore, they and or their families bear the costs of health care services.

### 3.2 Health status at baseline

Most male patients reported no health issues before they developed LF. Some of the definitions of ‘health’ were subjective: two equated health with having never gone to a hospital before, and one described himself as healthy because he had “only piles (hemorrhoids).” Among the three males that reported illness, one had visual impairment, another had hearing impairment, and the third had typhoid fever before the onset of LF. Three of the four female respondents reported suffering from either febrile illness, high blood pressure, peptic ulcer disease, chest, or back pain. The remaining participants reported that they were generally healthy prior to the onset of LF.

### 3.3 Knowledge and perception of Lassa fever

There was little knowledge about LF in the communities and among most respondents before their own experience of the illness. Typically, LF could not be differentiated from other febrile illnesses, particularly malaria, and most had never heard of its occurrence, even in other communities. Two people reported that they only found out LASV was transmitted from rats during the outbreaks, learning that the rodents differed from the ones commonly found in their communities. Most respondents sought care at the onset of infection from health facilities around them (chemists, clinics, dispensaries, and hospitals). Many were initially treated nonspecifically for febrile illnesses, and some were diagnosed with typhoid fever and treated. Some of the symptoms experienced by respondents were severe and persistent fever, headaches, body aches, dizziness and weakness. A few bled from the ears and mouth, some had hemoptysis and were subsequently referred to bigger hospitals.

Participants reported that opinions differed in communities over the cause of morbidities and mortalities during outbreaks of LF. While some pursued medical care, others attributed LF to witchcraft and sought traditional remedies, which created disharmony in the community. Disease surveillance teams conducted door-to-door testing but some interviewees reported that some members of the community refused the tests while others were in denial when the results confirmed that they were infected with LASV:

“Sometimes health personnel will come and pick you; some will agree to be taken, some will not agree, even if they will die. Some will go and die there; some will even die on the way; honestly, we lost a lot of people. We have suffered from this disease… About 7 people died in a house; from the General Hospital, they came and said it was LF, but they (members of the community) said it was not LF. At the beginning, we thought it was due to unbelief; later, some said it was fear mongering; others said Lassa is not real, like in the case of Corona virus infections where some believed while others did not.” (IS’s Caregiver, Male Respondent).

The increasing number of morbidities or mortalities within the community became a major source of concern, including to those who initially attributed their health condition to fate or witchcraft. Those who initially rejected the idea of going to a hospital with their sick family members were now willing to seek modern medical care. A third of respondents reported that they and other family members had symptomatic LF requiring hospitalization, which changed their perception on the nature of the ill-health they were experiencing:

“Previously, we saw it as a normal disease, but when we started losing our relatives at home, today one person is taken to the hospital and you will hear that God has called him to rest, some will survive some will not… No, it really shook us” (RY, Female respondent).

A male and female interviewee each lost a spouse, another lost a sibling, and a child survivor lost his mother during the outbreak. A survivor recounts his understanding of why his sibling died before he became infected with Lassa fever:

“I saw it as a joke and not true; when my brother died, I said ‘it is a lie, his time for dying has come, and that is why he died’, I did not agree. When it happened to me, I then agreed that Lassa truly exists. Finally, I agree Lassa is exists.” (HZ, Male Respondent).

A third of the respondents similarly reported that other families in the community were infected with LASV and also faced losses. It was reported that in one community, 20 people died before the outbreak was reported to the health authorities. Interviewees recalled families losing several members within a short period of time, which seemed to change people’s perceptions about the reality of LF and led to a greater willingness to undergo diagnostic tests.

### 3.4 Stigma with Lassa fever infection

Among respondents, we found several cases of stigma at the family, community, and intercommunity levels. A male survivor was deeply affected because his wife left him, taking their two children with her due to the fear of infection. He reported that other family members and neighbors also avoided him. Many participants described the feeling of being isolated, unlike in other illness scenarios where people receive social support, including hospital visitation. A widow who is a survivor but lost her husband to LF described her experience:

“There was stigmatization. They stopped people from entering our houses. Till today, people I relate with could not come to my house to greet me (over husband’s death and her ill-health). They are just avoiding you so that they will not be infected. They believe that if they just touch you, they will contact it.” (HM, Female respondent).

One respondent also explained how sections of a community without incidence of LF will not console families in an affected part once LF was announced as the cause of death. Stigma was reported to be problematic in neighboring communities:

“…you can ask anyone in this community, many people died, even in neighboring communities. It got so bad that if someone died, finding who will bury him became a big problem. Everyone was running.” (IS’s Caregiver, Male Respondent).

Another instance of community stigma was a male survivor discovering that people tried to discourage his fiancée from marrying him due to his history of LF and neighbors making unkind remarks to survivors when they lost several members of their family.

Another respondent also described discrimination by healthcare providers at the hospital when patients presented with LF signs:

“We stayed for more than 2 h in the car; no one checked on me… My blood sample was not collected until they took me to the Lassa camp. You see, there is a problem there. Until I was admitted, I spent about three hours before my blood sample was collected for testing.” (HI, Female Respondent).

In isolation, she reported great bodily weakness; she was unable to drink water without assistance and had to use the restrooms outside the facility with no staff in sight. She was not allowed the use of her mobile phone, could not tell the time, and her experience was terribly alienating.

### 3.5 Stigma due to hearing loss

All respondents indicated that they experienced hearing impairment after recovering from LF, mostly unilateral with a few cases of bilateral hearing loss. One patient suffered brain injury and several others experienced tinnitus after recovery from LF. Some described instances of isolation and labeling in their communities, which are common forms of enacted stigma.

The sequelae of hearing impairment affected the family relationships of a few respondents. One woman described how her communication was affected.

“…if someone talks to you and you could not hear, they get upset.” (HI, female).

Similarly, her children did not understand her hearing issues and would become angry, believing that she ignored them when she did not respond. Another survivor’s wife would sometimes want to dismiss the conversation but he would encourage her and reassure himself:

“If she talks to me and I did not hear, she will say ‘that’s all’ (terminating the conversation). I will tell her ‘No, do not give the devil chance’ and say that ‘all I know is that only God can heal me.’ I use words that will encourage me.” (SI, Male Respondent).

Some respondents reported that relatives, neighbors and community members avoided them or insulted them due to their hearing loss. One of the respondents challenged his neighbors and relatives who avoided him since he became ill:

“…I tell them that ‘since I got sick, you have avoided me’ and they respond by saying ‘no, if we come and speak to you ten times, you will not comprehend. That is what discourages us.’” (HZ, Male Respondent).

Another respondent admitted he suffered socially and emotionally due to hearing loss. He described his situation thus:

“They might do something, and I will not hear. So many things made me angry—some people were mocking me; when they talk and I did not hear them, they will insult me. Since I am human, I felt bad.” (SI, Male Respondent).

There were some associated economic losses resulting from the stigma that participants experienced. A few business owners explained how their hearing difficulty affected communication with customers and that they relied heavily on former clients to bring in business. One previously employed respondent reported stigma from his colleagues:

“…. they used to challenge me to go and find medicine because when they speak to me, I do not hear. I said it is from God, and problems that come from God can only be solved by God.” (AS, Male respondent).

An incident of self-stigma was also reported where a female respondent, who upon discharge from hospital decided to discontinue her trade of frying bean cakes, for fear of avoidance and mockery by the community. Mockery in the form of labelling and name calling was identified many times as a barrier for survivors returning to work:

“Yes, they will say hey, you deaf man, or please touch that deaf man for me. The moment this happens to you, just know that your name has changed.” (IS’s Caregiver, Male Respondent).

Several respondents expressed sadness over their experiences of stigma due to LF and hearing loss, and the psycho-social and economic costs of their condition.

Some respondents reported that they were not stigmatized. We discovered that there were no reported cases of stigma in places of worship. In fact, a respondent reported that his pastor was the first to visit him when he returned home from hospitalization for LF. He was also warmly welcomed during his first church service after the illness. Similarly, a respondent with hearing loss received help to understand the messages at the mosque that were not understood. A community leader described how the entire community welcomed him back after he was discharged, and a caregiver reported how a child survivor with hearing loss was well taken care of by male siblings when their mother died from LF infection.

### 3.6 Prevention of Lassa fever virus infections

Pertaining to their perceptions on the prevention of LF in their communities, some participants believed that the government could help improve prevention by sustained awareness and sensitization, including performing house-to-house sensitization on LASV transmission. There were suggestions for the provision of financial resources for treatment, public health, and social amenities that rural facilities lack and could benefit LF patients. A suggestion for vaccines against LASV was given by one as a means of securing the health of people who may not easily change their lifestyles. They also suggested that communities should comply with the directives of health personnel and report illnesses in a timely manner to ensure a proper diagnosis and early treatment. Another respondent advocated for isolation of affected persons, by saying that.

“…They should allow such a person to get the treatment he needs; it is not necessary that people should come and see you because in the process the other person can also get infected.” (HI, female respondent).

The need for confidentiality in managing cases of LF and proper counselling of patients to avert trauma after receiving the news abruptly was also suggested by the respondents.

## 4 Discussion

Our research findings show that there was little knowledge about LF and the severity of the illness in the affected communities when they experienced initial outbreaks, which led to a delay in seeking for help. There was also general fear of LF in communities due to its high morbidity and the fatal outcomes of some cases, leading to generalized stigma of areas with high incidence and alteration of customary intra- and inter-community support during bereavements and burials. Our study also shows that some patients who developed HL following LASV infection experienced double stigma both due to the infection itself and the subsequent hearing loss. Most commonly, persons in the community alienated both survivors and uninfected family members. A systematic review and meta-analysis by Yuan et al. (16) estimated the prevalence of stigma to be 34% among patients who had infectious diseases, and Usuwa et al. (2) similarly found that 32.2% of respondents in Ebonyi State, Nigeria said they would stigmatize survivors of LASV infections, which support our finding on societal stigma after LASV infection and hearing loss.

We found that prior to outbreaks, there was little knowledge of LF and the routes of LASV transmission in the communities. This is in keeping with a study that found that only 15.4% of participants from rural areas had good knowledge of Lassa fever (19) and another study that showed that the majority of health workers surveyed had inadequate knowledge of Lassa fever (20). However, another study showed that healthcare workers generally had fair knowledge and a good perception of Lassa fever (21). Rats are normally found in rural communities/domestic spaces, where they are sometimes eaten (22) and are not known as a source of morbidity or mortality. Community members often mistook LF symptoms for those of other febrile illnesses; therefore, there was poor health-seeking behavior and delays in diagnosis and treatment. The outbreaks caused significant morbidity and mortality that generated fear, which contributed to the stigmatization of patients, creating social disharmony.

Enacted stigmatization was experienced by some of the respondents in our study. However, only one person reported self-stigma, and one respondent experienced discrimination from a health provider. Denial of results or avoiding diagnostic tests reported in communities during the outbreaks attests to stigma as a social problem that can affect prevention and treatment. Stigma was identified as the ‘third epidemic’ hampering the prevention, treatment and management of HIV and AIDS (23, 24). Furthermore, a recent systematic review and meta-analysis revealed that 37% of people with infectious diseases from low- or middle-income countries experience stigma (16). Experiences of stigma and discrimination among the patients with LF and HL that participated in this study show that they were bereft of the psychosocial support they needed to recover and reintegrate into society, increasing their pain and otherness. This may have discouraged others with similar symptoms from seeking appropriate help for fear of experiencing the same fate, thereby increasing the potential for disease spread within the communities.

Some respondents experienced economic losses on account of their infection with LASV and the associated HL. However, they did not associate their economic losses with stigmatization. One attributed the loss of trade to his inability to procure newer equipment to work and another could not continue her trading because of the tinnitus that disturbed her hearing. This finding contrasts to those of Sharac et al. (25), who reported that mental illness from stigma/discrimination impacted negatively on employment, income, public views about resource allocation, and healthcare costs. This may be because some of their clients were unaware of their health condition as indicated by one of the respondents.

Some patients with hearing loss faced challenges navigating daily social interactions with family members and other associates, who are unable to communicate effectively with them, evoking emotional pain as they experienced alienation when communication could not be effectively achieved. They also experienced verbal abuse as some were labelled “deaf.” Marginalization of people with hearing loss is common because their unique communication and accessibility needs often highlight their “otherness” from people without hearing challenges (15). This explains why some of the respondents in our study who experienced stigma due to their hearing deficit felt the strain and pain of poor communication in their relationships, which needs to be addressed to restore and improve empathy and acceptance of changes by family and community members when communicating with patients with HL. Moreover, negative attitudes, misinformation, and institutional practices have been identified as drivers of stigma in Africa (26). This raises the need for a public health framework that includes a more robust information and education plan to guide: (1) the prevention and timely treatment of LF in all communities in the country; (2) community sensitization and awareness building that addresses stigma and provides opportunities to learn sign language to facilitate communication and reduce the isolation of patients. Lassa fever patients may require psychological rehabilitation to prevent other mental health consequences of stigmatization.

Nigeria has a surveillance system for infectious diseases, including LF, which is supported and strengthened by the Nigeria Centre for Disease Control (NCDC). Surveillance training is provided for public health officers at national and state levels and sensitization is conducted through workshops for healthcare workers; public media, such as radio, television, poster, and social network services, are used to sensitize the general public. There is also a sample transportational system and a laboratory network for the in-country diagnosis of LF. The NCDC provides regular situation reports on LF and clinical practice guidelines. There are also defined threshold levels and criteria for the activation of emergency operations centers across the country. Specialized centers for the treatment of LF are an integral part of the system for the control of the disease (27). However, there is no active mode of management for LF-induced hearing loss. Conservative methods of management include hyperbaric labyrinthine vasodilators (e.g., nicotinic acid), hyperbaric oxygen, and carbogen therapy (28) to enhance the oxygen pressure/perfusion to the inner ear. Steroids and low molecular weight dextran have also been used. However, there is no clear evidence on the efficacy of these methods of treatment. The effect appears to be similar to that seen in the management of idiopathic hearing loss where most improved cases are believed to be spontaneous rather than as a result of treatment. Rehabilitation using hearing-aids and, in those with severe to profound hearing loss, cochlear implants is recommended (29, 30).

The strength of this study is that it explores stigma and discrimination in patients with both LF and HL, which, based on our knowledge, has not been previously done. Our study had limitations. The study was conducted in communities in only one State in Nigeria; therefore, the findings may not be generalizable to other settings. However, it provides in-depth insight into the perceptions and experiences of persons in the studied communities. We recommend that future studies should include more communities, healthy members of the community, and health workers to triangulate the findings and provide more robust evidence on the stigma associated with HL arising from Lassa fever.

## 5 Conclusion

Persons with LF who develop hearing loss may experience double stigmatization both due to the illness and its sequelae. Hearing loss poses a social challenge to both LF survivors who develop hearing complication and their family members, especially during daily interactions, due to the frustrations and difficulty they face when attempting to communicate. An integrated public health approach to address LF, its sequelae of HL, and the associated stigma is required.

## Data availability statement

The original contributions presented in the study are included in the article/supplementary material, further inquiries can be directed to the corresponding author.

## Ethics statement

The studies involving humans were approved by Jos University Teaching Hospital Research Ethics Committee (Approval number: JUTH/DCS/ADM127/XXVI/669 & NHREC/JUTH/05/1022). The studies were conducted in accordance with the local legislation and institutional requirements. Written informed consent for participation in this study was provided by the participants’ legal guardians/next of kin.

## Author contributions

KB: Conceptualization, Methodology, Supervision, Writing – original draft, Writing – review & editing. EA: Formal Analysis, Writing – original draft, Writing – review & editing. CW: Conceptualization, Formal analysis, Methodology, Project administration, Writing – original draft, Writing – review & editing. DS: Writing – original draft, Writing – review & editing. IM: Writing – original draft, Writing – review & editing. IT: Writing – original draft, Writing – review & editing. NM: Supervision, Writing – original draft, Writing – review & editing. CY: Writing – original draft, Writing – review & editing. TM: Funding acquisition, Supervision, Writing – original draft, Writing – review & editing. SW: Conceptualization, Funding acquisition, Methodology, Project administration, Resources, Supervision, Validation, Writing – original draft, Writing – review & editing. SP: Conceptualization, Funding acquisition, Methodology, Project administration, Resources, Supervision, Validation, Writing – original draft, Writing – review & editing. NS: Conceptualization, Data curation, Formal analysis, Funding acquisition, Methodology, Project administration, Resources, Supervision, Validation, Writing – original draft, Writing – review & editing.
